# Generation and Characterization of hiPS Lines from Three Patients Affected by Different Forms of *HPDL*-Related Neurological Disorders

**DOI:** 10.3390/ijms251910614

**Published:** 2024-10-02

**Authors:** Matteo Baggiani, Devid Damiani, Flavia Privitera, Stefania Della Vecchia, Alessandra Tessa, Filippo Maria Santorelli

**Affiliations:** 1 Molecular Medicine for Neurodegenerative and Neuromuscular Diseases Unit, IRCCS Fondazione Stella Maris, Via dei Giacinti 2, Calambrone, 56128 Pisa, Italy; matteo.baggiani@fsm.unipi.it (M.B.); flavia.privitera@fsm.unipi.it (F.P.); stefania.dellavecchia@fsm.unipi.it (S.D.V.); alessandra.tessa@fsm.unipi.it (A.T.); 2 Department of Neurosciences, Psychology, Drug Research and Child Health (NEUROFARBA), University of Florence, Viale Pieraccini, 6, 50139 Florence, Italy

**Keywords:** HSP, SPG83, HPDL, hiPS lines, reprogramming protocol, neural differentiation

## Abstract

Hereditary spastic paraplegias are rare genetic disorders characterized by corticospinal tract impairment. Spastic paraplegia 83 (SPG83) is associated with biallelic mutations in the *HPDL* gene, leading to varied severities from neonatal to juvenile onset. The function of HPDL is unclear, though it is speculated to play a role in alternative coenzyme Q10 biosynthesis. Here, we report the generation of hiPS lines from primary skin fibroblasts derived from three SPG83 patients with different *HPDL* mutations, using episomal reprogramming. The patients’ clinical characteristics are carefully listed. The hiPS lines were meticulously characterized, demonstrating typical pluripotent characteristics through immunofluorescence assays for stemness markers (OCT4, TRA1-60, NANOG, and SSEA4) and RT-PCR for endogenous gene expression. Genetic integrity and identity were confirmed via Sanger sequencing and short tandem repeat analysis. These hiPS cells displayed typical pluripotent characteristics and were able to differentiate into neocortical neurons via a dual SMAD inhibition protocol. In addition, HPDL mutant neurons assessed via long-term culturing were able to achieve effective maturation, similarly to their wild-type counterparts. The HPDL hiPS lines we generated will provide a valuable model for studying SPG83, offering insights into its molecular mechanisms and potential for developing targeted therapies.

## 1. Introduction

Hereditary spastic paraplegias (HSPs) comprise a family of heterogeneous rare genetic diseases, sharing the common characteristics of corticospinal tract structure and function impairment. HSPs can present as pure forms, in which patients present weakness and spasticity in the lower limbs, and complex ones, in which paraparesis can be accompanied by such other neurological and extra-neurological manifestations as cerebral palsy, peripheral neuropathy, amyotrophic lateral sclerosis, and cerebellar ataxia [1,2]. The genetic etiology of HSPs is intricate, and approximately 90 different genes have been associated to date [3,4].

One of the most recently identified genes is 4-HydroxyPhenylpyruvate Dioxygenase-Like (*HPDL*), a single-exon gene encoding a protein that has been associated with rare, progressive childhood-onset movement disorders, with a broad clinical spectrum ranging from severe neurodevelopmental disorder with progressive spasticity and brain white matter abnormalities (NEDSWA phenotype MIM #619026) to juvenile-onset HSP (SPG83, MIM #619027) [5,6,7,8,9,10,11].

The role of the protein encoded by the *HPDL* gene is still unclear. *HPDL* is the only mammalian paralogue of the 4-HydroxyPhenylpyruvate Dioxygenase gene (*HPD,* MIM*609695), which participates in the tyrosine catabolic pathway, but the gene seems to have a different function [5,6]. *HPDL* encodes a 40 kDa polypeptide containing a mitochondrial targeting sequence, two vicinal oxygen chelate (VOC) domains, and three iron-binding sites. Functional experiments confirmed HPDL localization in mitochondria and its wide expression in most organs, especially in the brain, in which glia have been suggested as the major source of *HPDL* transcripts [6]. Besides its nominal enzymatic function as “dioxygenase”, HPDL activity has been speculatively associated with oxidative phosphorylation [9], apoptosis [6], and both cell proliferation and differentiation [12]. Nonetheless, the precise function of the protein so far remains obscure. To shed light on these aspects, mouse and zebrafish (*Danio rerio*) models have been generated, suggesting that the protein may have a role in neurodevelopment. *Hpdl*^−/−^ mice displayed smaller brain sizes with apoptosis in cortical tissue and epilepsy, resembling features observed in children with pathogenic gene variants [6]. Unfortunately, perinatal lethality, affecting the totality of mutant animals, did not allow a proper longitudinal study, thereby impeding crucial characterization of phenotypes that arise and progress in adult animal life due to accumulation of cellular damage through neurodegenerative mechanisms. Transient *hpdl* zebrafish knock-down mutants generated with morpholino oligonucleotides lacked obvious morphological differences with control larvae but showed an impaired response to stimulation and impaired locomotion [10]. The presence of motor impairments, mimicking milder traits of the human disease, is interesting but the transient nature of the model again does not allow the progression of the disease to be monitored over time [10].

Adding to this, in vitro studies in non-neuronal cell models did not show a strong presence of mitochondrial dysfunction, while impairment of cellular respiration has been described in neural cell lines and brain tissues [9]. In this scenario, a recent study using oxy-metabolomics proposed that HPDL could play an important role in an alternative coenzyme Q10 (CoQ10) biosynthetic pathway, uniquely responsible for the conversion from the known tyrosine catabolite 4-hydroxyphenylpyruvate to 4-hydroxymandelate [13].

In this work, we obtained cultured skin fibroblasts from three SPG patients carrying biallelic mutations in the *HPDL* gene, we generated human induced pluripotent stem (hiPS) cells through an optimized episomal reprogramming protocol, and we demonstrated effective differentiation of mature neocortical neurons. These cells, finely characterized following standard guidelines, will be useful in the future to shed light on both developmental and degenerative aspects of neurological disorders caused by the absence of HPDL protein in SPG83 patients.

## 2. Results

Three HPDL hiPS lines were generated starting from human dermal fibroblasts (HDFs) deriving from three SPG83 patients carrying homozygous (Patient 1) or compound heterozygous mutations (Patients 2 and 3) in the coding sequence of the gene *HPDL*. In particular, Patient 1 carried a biallelic missense mutation resulting in a Ser49Arg substitution in the VOC1 domain of the HPDL protein; Patient 2 instead carried a proximal deletion, resulting in a frameshift starting from aminoacidic position 86, and a missense mutation generating Ile266Thr in the VOC2 domain of HPDL; Patient 3 carried two different missense mutations, resulting in Phe31Leu substitution in the VOC1 domain and a Gly278Ser switch in the VOC2 domain of the HPDL protein (a more detailed description of patients’ features are reported in Table 1 and Table S1).

### 2.1. Reprogramming and Characterization

**Figure 1:**
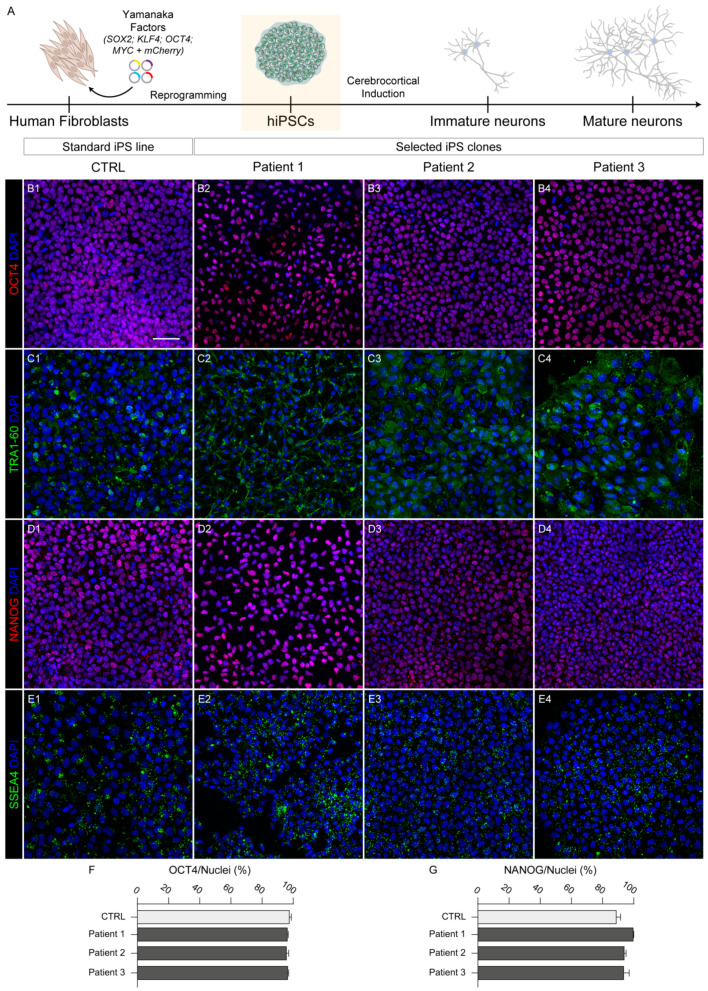
Generation of HPDL mutant hiPS cell lines deriving from three SPG83 patients. (**A**) Schematic representation of workflow starting from human fibroblasts reprogrammed into hiPS cells, and then cortically induced in neurons. (**B1**–**B4**, **C1**–**C4**, **D1**–**D4**, **E1**–**E4**) Representative confocal images with stemness markers OCT4, TRA-1-60, NANOG, and SSEA4, respectively, in CTRL and HPDL hiPS cells. All nuclei were stained with DAPI. Scale bar: 50 μm. (**F**) Bar plot indicating the ratio of OCT4-positive cells on total nuclei in CTRL and HPDL cell lines (counted cells: CTRL, N = 1892; Patient 1, N = 778; Patient 2, N = 971; Patient 3, N = 984). (**G**) Bar plot indicating the ratio of NANOG-positive cells on total nuclei in CTRL and HPDL cell lines (counted cells: CTRL, N = 1807; Patient 1, N = 864; Patient 2, N = 1481; Patient 3, N = 1766).

HDFs were reprogrammed via transfection of four episomal vectors expressing human *OCT4*, *SOX2*, *LIN28*, *L-MYC*, *KLF4*, and shRNA for *TP53* (Figure 1A), obtaining hiPS colonies after about 3–4 weeks in culture.

**Figure 2:**
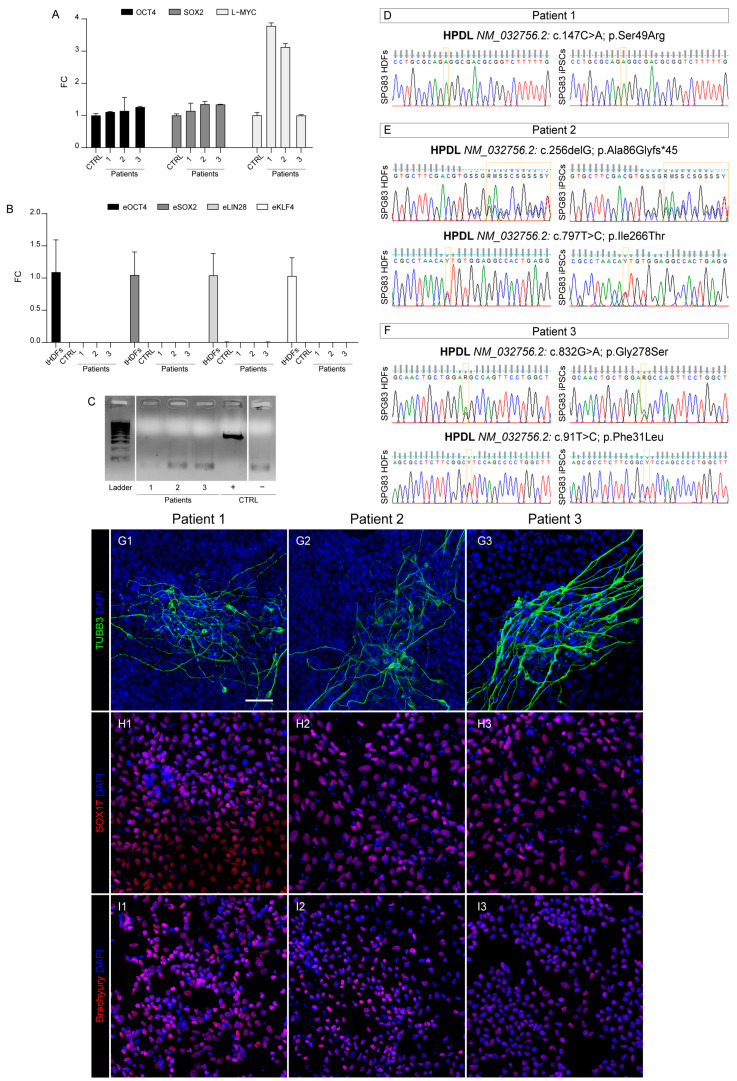
Characterization of three HPDL hiPS cells derived from patients’ primary cell lines. (**A**) Bar plot showing the same or higher expression of OCT4, SOX2, and L-MYC genes in our generated hiPS cells compared with CTRL ones. (**B**) Bar plot shows the total absence of eOCT4, eSOX2, eLIN28, and eKLF4 episomal gene expression in CTRL and our HPDL hiPS cells compared with tHDFs. (**C**) Gel image showing the lack of mycoplasma contamination in our patient-derived hiPS cells. (**D**–**F**) Electropherograms showing that the parental HDF genotype was maintained in the corresponding hiPS lines. (**G1**–**G3, H1**–**H3, I1**–**I3**) Representative confocal images with stemness markers b3-tubulin (TUBB3), SOX17, and Brachyury, respectively, in differentiated cells derived from all HPDL hiPS lines. All nuclei were marked with DAPI. Scale bar: 50 μm.

HPDL hiPS clones displaying the best morphology features, namely well-defined edges and a high nuclear/cytoplasmic ratio (Figure S1A–C), were isolated and propagated for at least seven passages before further characterization. In order to verify the actual stemness, all three HPDL patient-derived hiPS lines were verified by both immunofluorescence assay for stemness markers such as OCT4, TRA1-60, NANOG, and SSEA4 (Figure 1B–E), quantifying the ratio of OCT4- and NANOG-positive cells (Figure 1F,G), and by qRT-PCR, evaluating expression of *OCT4*, *SOX2*, and *L-MYC* (Figure 2A), in comparison with the CTRL line.

Moreover, to verify that reprogramming plasmids did not integrate into the hiPS cell genomes, we quantified expression of episomal genes *eOCT4*, *eSOX2*, *eLIN28*, and *eKLF4* by qRT-PCR. As a positive control, we used RNA taken from an HDF line harvested right after plasmid transfection. Consistently, episomal transcripts were undetectable in pluripotent cell lines (Figure 2B), confirming that the stemness properties of our HPDL hiPS lines derived from endogenous genes.

### 2.2. Mycoplasma

Possible contamination from mycoplasma can sometimes occur in cell culture facilities, constituting a modifying factor capable of altering cellular characteristics such as viability, proliferation, differentiation potential, etc. Our PCR-based test ruled out this possibility, showing an absence of mycoplasma DNA in all selected iPS clones, as reported in Figure 2C.

### 2.3. Genome Identity

Towards the end of verifying the correct genomic identity of our hiPS cells, we tested for the presence of mutations of SPG83 patient-derived hiPS cells by using PCR on genomic DNA and subsequent Sanger sequencing, comparing them with corresponding HDF parental lines (Figure 2D–F). In addition, short tandem repeat (STR)-based fingerprinting analysis was used to ascertain the genomic sameness between HPDL hiPS cells and parental HDFs. In all cases, microsatellites from 18 different genetic loci showed a perfect match among the patient lines (Figure S1D–F).

Moreover, to rule out the unlucky event of genomic rearrangements [14,15], we performed a detailed genomic analysis by array-CGH, analyzing mutant hiPS cells and their HDF parental lines and evaluating the genome integrity of our clones. The results of this comparison clearly showed the presence in pluripotent cells of the same polymorphisms found in parental HDFs, demonstrating that genomic stability was maintained upon reprogramming procedures (Figure S2).

### 2.4. Pluripotency Capability

Finally, to demonstrate the pluripotent capacity of our HPDL hiPS lines, we performed a differentiation protocol aimed at obtaining the three different embryonic germ layers (ectoderm, endoderm, and mesoderm). For this purpose, hiPS cells were cultured in trilineage differentiation medium in three different conditions. In the first case, we let cells differentiate spontaneously, as no morphogens were added in the medium. These conditions were permissive for differentiation of ectodermal cells, as testified by rapid emergence of neuronal cells (positive for marker TUBB3; Figure 2G). In order to acquire an endodermal or mesodermal identity, specific conditions were instead applied. In particular, cells positive for endodermal marker SOX17 (Figure 2H) were generated via administration of Wnt pathway activator CHIR99021 (5 µM) for 1 day, followed by TGF-beta activator activin A (100 ng/mL) for 3 days. Instead, mesodermal differentiation was induced by adding CHIR99021 (5 µM) for just 2 days, obtaining cells positive for the mesodermal marker brachyury (Figure 2I).

### 2.5. Neocortical Differentiation

**Figure 3:**
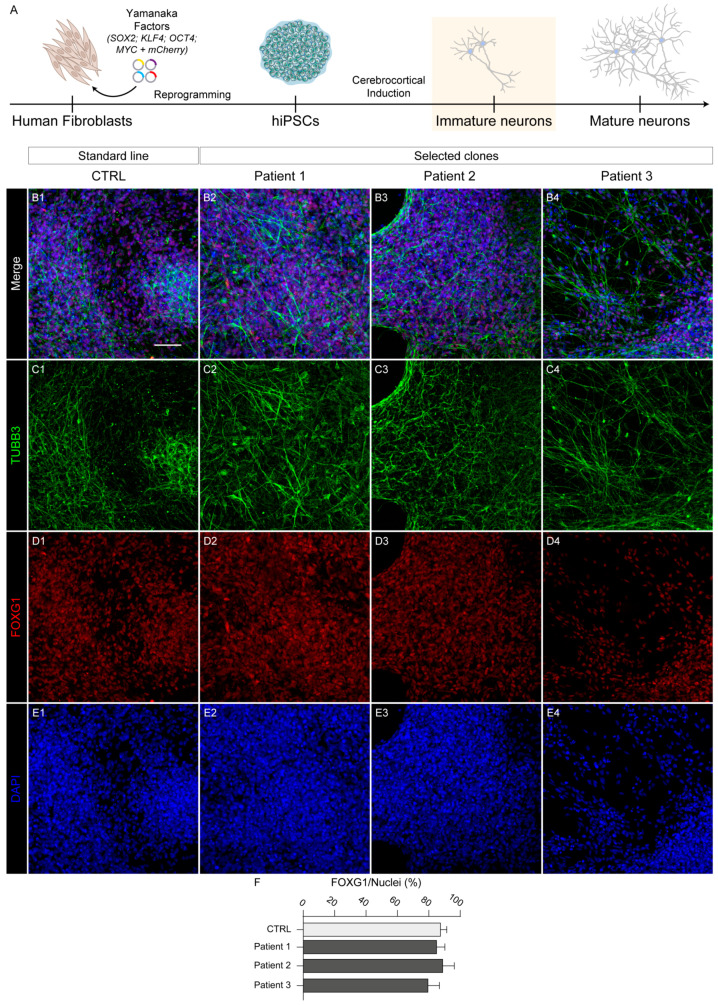
Early neuronal differentiation of three HPDL patient-derived hiPS lines. (**A**) Schematic representation of neuronal differentiation protocol to obtain neocortical neurons, starting from human fibroblast-reprogrammed hiPS cells. (**B1**–**B4, C1**–**C4, D1**–**D4, E1**–**E4**) Representative confocal images with neuronal markers TUBB3 and FOXG1 in CTRL and all HPDL immature neurons. All nuclei were marked with DAPI. Scale bar: 50 μm. (**F**) Bar plot indicating the ratio of FOXG1 positive cells on total nuclei in CTRL and HPDL cell lines (counted cells: CTRL, N = 4951; Patient 1, N = 3658; Patient 2, N = 3404; Patient 3, N = 3991).

As previously described, SPG83 patients harbor different type of *HPDL* mutations and they can present heterogeneous clinical symptoms. Nevertheless, the characteristics of corticospinal tract structure and functional impairment are common to all patients as hallmarks of spastic paraplegia. In this context, we verified the potential of the generated HPDL hiPS lines to differentiate in neocortical neurons. In particular, we performed a dual SMAD inhibition protocol [16] on each hiPS line (Figure 3A), evaluating the outcome of cellular differentiation with morphological and immunofluorescence assays.

To confirm the neocortical identity of differentiating cultures, we stained cells for both neuronal and telencephalic specific markers (TUBB3 and FOXG1, respectively; Figure 3B–E). The majority of differentiated cells showed positivity for both markers, demonstrating specific, homogeneous, and efficient corticopoiesis in both control and HPDL mutant cultures. In particular, extensive quantification of telencephalic cells (defined as FOXG1^+^/total nuclei ratio; Figure 3F) resulted rates of 88.8 ± 2.8%, 88.9 ± 3.7%, and 79.5 ± 3.2 for cells derived from Patient 1, Patient 2, and Patient 3, respectively. Considering that the same quantification performed on differentiated cultures from the control hiPS cells indicated a similar telencephalic proportion (88.2 ± 2.6%), no significant differences emerged from this analysis, demonstrating that HPDL mutant cell lines displayed physiological capability in terms of in vitro corticopoiesis.

**Figure 4:**
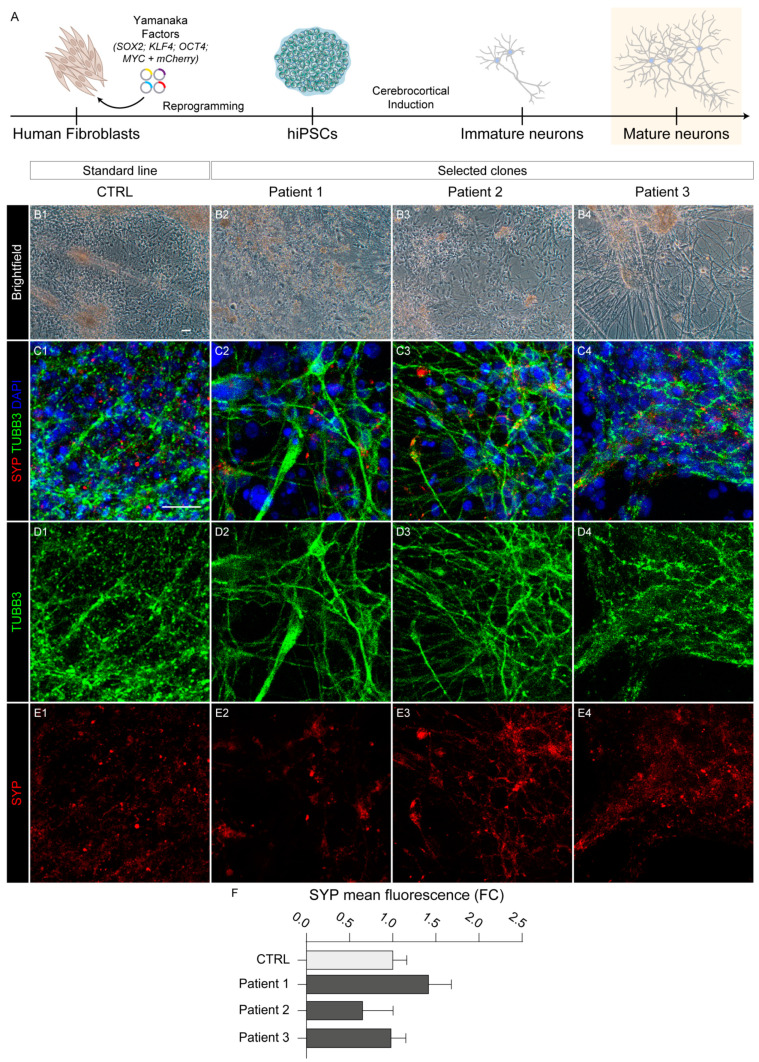
Late neuronal differentiation of three HPDL patient-derived hiPS lines. (**A**) Schematic representation of neuronal differentiation protocol to obtain neocortical neurons, starting from human fibroblast-reprogrammed hiPS cells. (**B1**–**B4**) Representative images of morphology from CTRL and all three HPDL hiPS cell-derived neurons. Scale bar: 50 μm. (**C1**–**C4, D1**–**D4, E1**–**E4**) Representative confocal images with neuronal markers TUBB3 and SYP in all CTRL and HPDL neurons. All nuclei were marked with DAPI. Scale bar: 20 μm. (**F**) Bar plot indicating the SYP mean fluorescence in CTRL and HPDL neurons (counted cells: CTRL, N = 2078; Patient 1, N = 3670; Patient 2, N = 2163; Patient 3, N = 7590).

In addition, we were able to culture differentiated cells for three months (Figure 4A), the stage at which neurons usually begin to express synaptic proteins and other maturation markers.

From the morphological point of view, HPDL mutant long-term cortical cultures showed well-differentiated neurons displaying long and intricated axonal networks (Figure 4B). Both wild-type and HPDL mutant neurons deriving from different patients displayed immunoreactivity for MAP2, RBFOX3/NeuN (Figure S3), and synaptic marker synaptophysin (SYP), demonstrating effective maturation of HPDL mutant neurons (Figure 4C–E). In addition, no significant differences emerged from comparison of the fluorescence of SYP between CTRL and HPDL cells (Figure 4F), demonstrating similar maturation capacity among different lines.

These first analyses confirmed that neurons differentiated from SPG83 patient-derived hiPS cells were able to maintain telencephalic specification and express markers of maturation with the same timing as their wild-type counterparts.

## 3. Discussion

From a clinical, cellular, and genetic point of view, HSPs represent a diverse group of rare heterogeneous disorders. These conditions are characterized by the progressive degeneration of the corticospinal tract, which has a key role in the nervous system for control of motor function. SPG83 is associated with autosomal recessive mutations in the *HPDL* gene. The biological importance of HPDL remains largely unknown, though it is hypothesized to play an important role in an alternative CoQ10 biosynthetic pathway. CoQ10 is essential for mitochondrial function and energy production, underscoring the potential impact of *HPDL* mutations on cellular metabolism.

The generation of hiPS cells through the reprogramming of somatic cells from patients carrying various pathological genetic variants provides a unique and valuable resource for studying the mechanisms underlying both developmental and degenerative aspects of neurological disorders. This approach is especially critical for SPG83, since no in vivo model has been able to effectively replicate the later stages of this neurological disease. The complexity of the human nervous system and the specific progression of SPG83 necessitate advanced cellular models as reliable platforms to explore comprehensively the pathophysiology of disease. From this perspective, the HPDL hiPS clones generated in this research showed several key properties. Notably, they exhibited high expression levels of stemness genes, overtaking in some instances those observed in ther CTRL hiPS cells. Additionally, reprogrammed cells maintained a perfect match with the parental HDFs in terms of type of mutation and genomic identity. Importantly, the pluripotent features of these hiPS cells were confirmed, indicating their potential to differentiate into various cell types from the three different embryonic germ layers. Finally, the proven capacity to reproduce HPDL mutant cortical tissue and to achieve the same maturation milestones as described for wild-type cells provides a reliable in vitro model for future research aimed at understanding the molecular and cellular mechanisms underlying the etiopathogenesis of SPG83, thereby paving the way for the potential development of targeted therapies. As a concrete example, experiments are currently ongoing in our lab to investigate the relationship between HPDL and CoQ10 in cortical tissue, trying to shed light on the role of this enzyme in the complex scenario of SPG83 disease.

## 4. Materials and Methods

### 4.1. Reprogramming of Skin Fibroblasts

HDFs obtained from patient skin tissue via punch biopsy were cultured and amplified in HDF medium (DMEM High Glucose, 10% Fetal Bovine Serum, L-Glutamine 2 mM, 100 U/mL PenStrep; Euroclone, Milan, Italy). Modifying the protocol described in the literature [17], our reprogramming protocol was used on the HDFs, nucleofecting in a cell suspension via Amaxa Nucleofector 2b (Lonza, Tampa, FL, USA), a mix of episomes for integration-free expression of human “Yamanaka’s factors” (L-MYC, LIN28, OCT3/4, SOX2, and KLF4). In particular, pCXLE-hUL (Addgene, Watertown, MA, USA; #27080), pCXLE-hOCT3/4-shp53-F + mCherry-2A-puro (gift from Ann Zovein, Addgene #74947), pCXWB-EBNA1 (Addgene #37624), and pCXLE-hSK (Addgene #27078, all gifts from Shinya Yamanaka), as we described previously [18]. Briefly, transfected HDFs (tHDFs) were cultured on Geltrex-coated dishes (Thermo Fisher Scientific, Waltham, MA, USA) with E6 medium containing 100 ng/mL FGF2 (named E7 medium), 0.5 mM sodium butyrate, 1 µM hydrocortisone, checking positive expression of mCherry after 2 days if the transfection was successful [19]. After at least 10 days, confluent cells were dissociated with trypsin (Euroclone) into single-cell suspension and kept in culture with E7 medium, adding 0.5 mM sodium butyrate for only 2 days, on Geltrex-coated dishes at 5.000 cells/cm^2^. From about 14 days onwards, hiPS “islands” appeared, resulting in a switch from E7 medium to Stemflex medium (Thermo Fisher Scientific) to support iPS cell survival and growth. hiPS clones with uniform flat and round-shaped morphology were picked in sterile conditions and propagated as individual cell lines.

### 4.2. Culture of hiPS Cells

hiPS cells were cultured at 37 °C in 5% CO_2_ on Geltrex-coated six-well plates in Stemflex medium, refreshing the medium every other day. Cells were passed every 5–7 days with ReLeSR™ Passaging Reagent (Stem Cell Technologies, Vancouver, BC, Canada), following manufacturer instructions.

### 4.3. Expression of Pluripotency Markers and Mutation Analysis

An All-prep DNA/RNA Mini Kit (Qiagen, Hilden, Germany) was employed to extract total genomic DNA and RNA from hiPS cells and parental HDFs, and a PrimeScript RT Reagent Kit (Takara Bio, Shiga, Japan) was used to synthesize cDNA from RNA. RT-qPCR on a Mic qPCR Cycler (Bio Molecular Systems, Upper Coomera, Australia) was utilized to determine OCT4, SOX2, and L-MYC expression of pluripotency markers in HPDL hiPS cells, normalizing values to expression of the established ACS-1019 hiPS line (ATCC, Manassas, VA, USA), named CTRL, via the 2^−∆∆CT^ method, using human GAPDH as a housekeeping gene. Moreover, to assess the possible presence of episomes, the relative expression of eOCT4, eSOX2, eLIN28, and eKLF4 was measured on the CTRL and HPDL hiPS lines, normalized on tHDFs. Primers used for qPCR reactions are listed in Table 2.

For immunofluorescence staining, hiPS cells were fixed with 4% formaldehyde for 12 min at RT and then permeabilized in PBS with 0.5% Triton X-100 for 10 min at RT. PBS with 5% normal goat serum (NGS) and 0.3% Triton X-100 was used as blocking solution for 1 h at RT and the cells were incubated with primary antibodies (Table 3) in antibody solution (PBS with 3% NGS and 0.2% Triton X-100) at 4 °C overnight. Cells were then washed three times with PBS and incubated in the same antibody solution with secondary antibodies (Table 3) for 1 h at RT. Nuclei were counterstained with DAPI (1 µg/mL; Merck, Darmstadt, Germany). All images were acquired with a Zeiss LSM 900 confocal microscope (Zeiss, Oberkochen, Germany).

### 4.4. Image Analysis

All images were processed with Fiji software v1.54f. In particular, the “Cell counter” plugin was used to quantify all nuclei positive for OCT4, NANOG, FOXG1, or DAPI. SYP fluorescence analysis was performed on cortical neurons double stained for SYP and TUBB3. The same threshold for TUBB3 fluorescence was applied on every image to select a region of interest (ROI). Then, each ROI was applied to the SYP channel to measure its mean fluorescence inside the neurons. Finally, all values were normalized to the CTRL condition. One-way ANOVA testing was used in all bar plots to analyze each comparison between CTRL and HPDL cells, and no significant difference was found.

### 4.5. Trilineage Differentiation

hiPS cells were cultured in trilineage differentiation medium (DMEM High Glucose, Glutamax 2 mM, 100 U/mL Pen/Strep, MEM-NEAA 1×, β-mercaptoethanol 0.1%; Thermo Fisher Scientific) to obtain differentiation into three different embryonic germ layers (ectoderm, endoderm, and mesoderm). In particular, cells spontaneously acquired markers for neuronal marker TUBB3, as previously reported [20]. Moreover, a modified version of the guidelines by Lam et al., 2014 [21] was employed to induce an endodermal identity, using 5 µM CHIR99021 (Stem Cell Technologies) for 1 day and then 100 ng/mL activin A (Stem Cell Technologies) for 3 days, and a mesodermal identity, using 5 µM CHIR99021 for 2 days. Endodermal-induced cells were positive for endodermal marker SOX17, while mesodermal-induced cells were marked with mesodermal marker brachyury. The medium was changed every day.

### 4.6. STR Analysis

The genomic identity between hiPS clones and parental HDFs was confirmed by gDNA typing with a PowerPlex 16HS multiplex STR system (Promega, Madison, WI, USA), including all 13 CODIS STR markers, amelogenin for gender determination, and Penta D and Penta E loci. The PCR products labeled with fluorescent dyes were detected with ABI-3500 Genetic Analyzer and data were analyzed with GeneMapper 5 (Thermo Fisher Scientific).

### 4.7. Genomic Analysis by Array-CGH

High-resolution whole-genome array-based comparative genomic hybridization (aCGH) analysis was performed on genomic DNA extracted from patient-derived hiPS cells, using the SurePrint G3 Human CGH Microarray 8 × 60 k (Agilent Technologies, Santa Clara, CA, USA), a dual-color array containing 60-mer high-quality probes with 41 Kb genome-wide median probe spacing. Copy number variants (CNVs) were analyzed and mapped using the Human Genome Assembly GRCh37/hg19. Slides were scanned using an Agilent G2600D Microarray Scanner (Agilent Technologies) and processed using Feature Extraction software (v12.1.0.3). Agilent CytoGenomics software (v5.0.2.5) was used to analyze the results with default settings. Imbalances with at least three consecutive probes with abnormal log2 ratios for deletions and at least four consecutive probes for duplications were included in the results. The Database of Genome Variants (http://dgv.tcag.ca, accessed on 25 September 2024), DECIPHER (https://www.deciphergenomics.org, accessed on 25 September 2024), and UCSC genome browser (https://genome.ucsc.edu, accessed on 25 September 2024) databases were used in the interpretation of the results. Coincident results were obtained when the same experiment was performed on fibroblast patient’s genomic DNA.

### 4.8. Mycoplasma Testing

PCR via a Mycoplasma PCR Detection Kit (ABM, Richmond, BC, Canada) was used to verify the absence of mycoplasma, according to the manufacturer instructions.

## 5. Conclusions

This study established and characterized three different patient-derived hiPS lines, each carrying distinct mutations in the *HPDL* gene, and confirmed their capability to effectively achieve cortical differentiation and neuronal maturation, similarly to standard lines. Combining multiple tools for modern preclinical research, this study underscores the importance of hiPS cell technology in advancing knowledge of rare neurological disorders and highlights the potential of patient-derived cells to offer new opportunities for personalized medicine.

## Figures and Tables

**Table 1:** Brief summary the characteristics of SPG83 donor Patients 1–3. All variants described refer to the Human Genome Assembly GRCh37/hg19. The GnomAD (https://gnomad.broadinstitute.org/, accessed on 25 September 2024) database was consulted in April 2024 to assess the allele frequencies.

	Patient 1	Patient 2	Patient 3
***HPDL* Variants**			
Mutations	NM_032756.2: c.147C>A(p.(Ser49Arg))	NM_032756.2: c.256delG(p.(Ala86Glyfs*45)) c.797T>C(p.(Ile266Thr))	NM_032756.2: c.91T>C(p.(Phe31Leu)) c.832G>A(p.(Gly278Ser))
Allele frequency (gnomAD)	0.001527%	0.0004896%/0.0008506%	Not reported
**Clinical Features and Age at Skin Biopsy**			
First manifestations	Developmental delay, neonatal seizures, spasticity in the lower limb	Motor delay and gait problem	Spastic gait
Age at onset (years)	Neonatal	1	4
Age at skin biopsy (years)	12	14	7
Disease severity/course	Intermediate phenotype	Intermediate phenotype	Mild phenotype

**Table 2:** Primers used for qPCR and genotyping reactions to verify the expression of stemness, housekeeping, and episomal plasmid genes and confirm the mutations.

Target	Size of Band	Forward/Reverse Primer (5′-3′)
*OCT4*	143 bp	Fwd: CCC CAG GGC CCC ATT TTG GTA CC
		Rev: ACC TCA GTT TGA ATG CAT GGG AGA GC
*L-MYC*	143 bp	Fwd: GCG AAC CCA AGA CCC AGG CCT GCT CC
		Rev: CAG GGG GTC TGC TCG CAC CGT GAT G
*SOX2*	80 bp	Fwd: TTC ACA TGT CCC AGC ACT ACC AGA
		Rev: TCA CAT GTG TGA GAG GGG CAG TGT GC
*GAPDH*	110 bp	Fwd: GGA AGG ACT CAT GAC CAC AGT
		Rev: GGA TGA TGT TCT GGA GAG CCC
*eOCT4*	124 bp	Fwd: CAT TCA AAC TGA GGT AAG GG
		Rev: TAG CGT AAA AGG AGC AAC ATA G
*eLIN28*	251 bp	Fwd: AGC CAT ATG GTA GCC TCA TGT CCG C
		Rev: TAG CGT AAA AGG AGC AAC ATA G
*eSOX2*	111 bp	Fwd: TTC ACA TGT CCC AGC ACT ACC AGA
		Rev: TTT GTT TGA CAG GAG CGA CAA T
*eKLF4*	156 bp	Fwd: CCA CCT CGC CTT ACA CAT GAA GA
		Rev: TAG CGT AAA AGG AGC AAC ATA G
*HPDL* A	785 bp	Fwd: CTTTCCGGAAGAAAGCGAGGAA
(Genotyping)		Rev: CCTCAGTCCCCCAAGCCCAA
*HPDL* B	661 bp	Fwd: TGCGCTGGTTCCACGACTGC
(Genotyping)		Rev: GCAGATGTTCCTCAGTTCTGTG

Regarding mutation analysis, the *HPDL* gene was amplified by PCR (PCR primers are listed in Table 2). The identity of mutations was confirmed via Sanger sequencing.

**Table 3:** Primary and secondary antibodies employed for immunofluorescence assay to stain stemness and differentiation markers.

Antibody	Dilution	Company, Cat #
Rabbit anti-OCT4	1:500	Abcam (Cambridge, UK), Cat #ab19857
Rabbit anti-Nanog (D73G4)	1:200	Cell Signaling Technology (Danvers, MA, USA), Cat #4903
Mouse anti-TRA-1-60	1:500	Cell Signaling Technology, Cat #4746
Mouse anti-SSEA4 (MC813)	1:500	Cell Signaling Technology, Cat #4755
Rabbit anti-FOXG1	1:500	Abcam, Cat #ab18259
Rabbit anti-synaptophisin	1:100	Cell Signaling Technology, Cat #36406
Mouse anti-TUBB3	1:1000	Abcam, Cat #ab7751
Rabbit anti-brachyury (D2Z3J)	1:500	Cell Signaling Technology, Cat #81694
Rabbit anti-SOX17 (D1T8M)	1:500	Cell Signaling Technology, Cat #81778
Mouse anti-MAP2	1:200	Sigma (St. Louis, MO, USA), Cat #ZMS1013
Rabbit anti-RBFOX3/NeuN	1:500	Millipore (Burlington, MA, USA), Cat #ABN78
Goat Anti-Mouse Alexa Fluor 488	1:500	Thermo Fisher Scientific, Cat #a11029
Goat Anti-Rabbit Alexa Fluor 555	1:500	Thermo Fisher Scientific, Cat #a21429

## Data Availability

Data sharing is not applicable to this article as no datasets were generated or analyzed during the current study.

## Supplementary Materials

The following supporting information can be downloaded at https://www.mdpi.com/article/10.3390/ijms251910614/s1.
