# Supplementary material for: Generation and Characterization of hiPS Lines from Three Patients Affected by Different Forms of HPDL-Related Neurological Disorders
Source: Int J Mol Sci. 2024 Oct 2;25(19):10614. doi: 10.3390/ijms251910614 (PMC11477155; doi:10.3390/ijms251910614)
Supplement: Supplementary file 1 [file ijms-25-10614-s001.zip › Supplementary Materials.pdf]

Supplementary Materials: Genetic identity of parental and hiPS lines

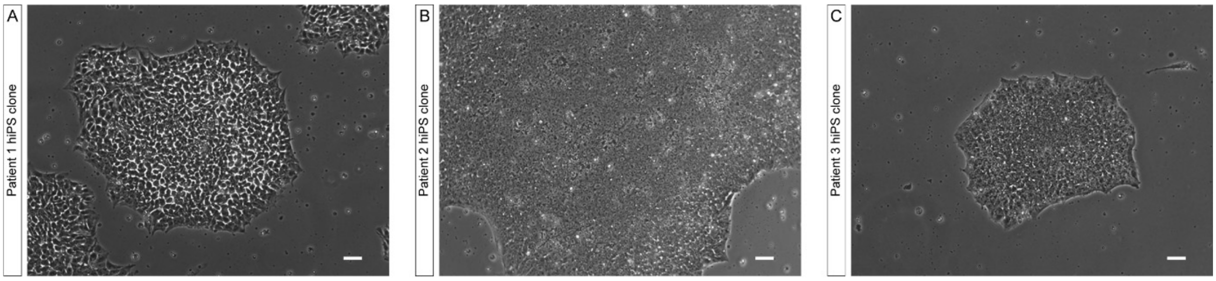

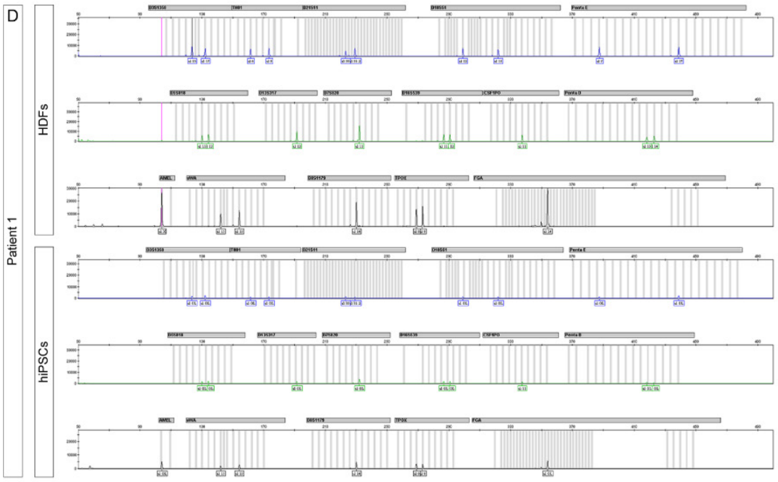

Sample File Name	Marker	Size
Patient 1 HDFs	D3S1358	124
Patient 1 HDFs	D3S1358	132
Patient 1 HDFs	TH01	162
Patient 1 HDFs	TH01	174
Patient 1 HDFs	D21S11	223
Patient 1 HDFs	D21S11	229
Patient 1 HDFs	D18S51	299
Patient 1 HDFs	D18S51	322
Patient 1 HDFs	Penta E	387
Patient 1 HDFs	Penta E	439
Patient 1 HDFs	D5S818	130
Patient 1 HDFs	D5S818	134
Patient 1 HDFs	D13S317	192
Patient 1 HDFs	D7S820	232
Patient 1 HDFs	D16S539	287
Patient 1 HDFs	D16S539	291
Patient 1 HDFs	CSF1PO	337
Patient 1 HDFs	Penta D	418
Patient 1 HDFs	Penta D	423
Patient 1 HDFs	AMEL	104
Patient 1 HDFs	vWA	142
Patient 1 HDFs	vWA	154
Patient 1 HDFs	D8S1179	230
Patient 1 HDFs	TPOX	269
Patient 1 HDFs	TPOX	273
Patient 1 HDFs	FGA	354

Sample File Name	Marker	Size
Patient 1 hiPSCs	D3S1358	124
Patient 1 hiPSCs	D3S1358	132
Patient 1 hiPSCs	TH01	162
Patient 1 hiPSCs	TH01	174
Patient 1 hiPSCs	D21S11	223
Patient 1 hiPSCs	D21S11	229
Patient 1 hiPSCs	D18S51	299
Patient 1 hiPSCs	D18S51	322
Patient 1 hiPSCs	Penta E	387
Patient 1 hiPSCs	Penta E	439
Patient 1 hiPSCs	D5S818	130
Patient 1 hiPSCs	D5S818	134
Patient 1 hiPSCs	D13S317	192
Patient 1 hiPSCs	D7S820	232
Patient 1 hiPSCs	D16S539	287
Patient 1 hiPSCs	D16S539	291
Patient 1 hiPSCs	CSF1PO	337
Patient 1 hiPSCs	Penta D	418
Patient 1 hiPSCs	Penta D	423
Patient 1 hiPSCs	AMEL	104
Patient 1 hiPSCs	vWA	142
Patient 1 hiPSCs	vWA	154
Patient 1 hiPSCs	D8S1179	230
Patient 1 hiPSCs	TPOX	269
Patient 1 hiPSCs	TPOX	273
Patient 1 hiPSCs	FGA	354

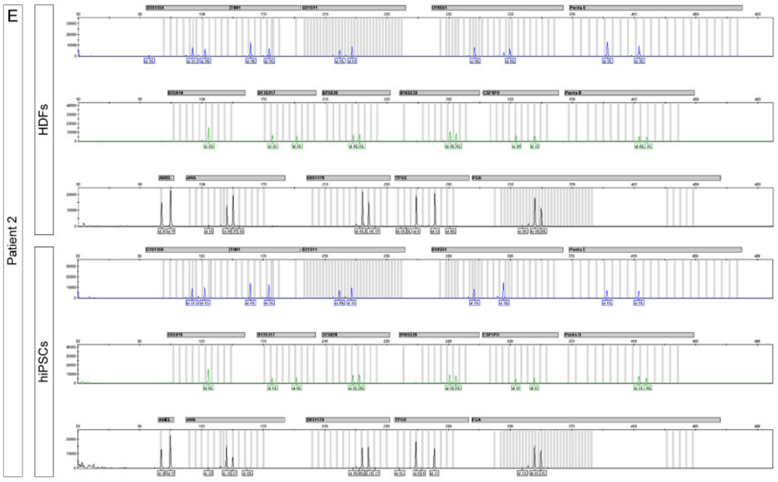

Sample File Name	Marker	Size
Patient 2 HDFs	D3S1358	124
Patient 2 HDFs	D3S1358	132
Patient 2 HDFs	TH01	162
Patient 2 HDFs	TH01	174
Patient 2 HDFs	D21S11	219
Patient 2 HDFs	D21S11	227
Patient 2 HDFs	D18S51	307
Patient 2 HDFs	D18S51	329
Patient 2 HDFs	Penta E	393
Patient 2 HDFs	Penta E	413
Patient 2 HDFs	D5S818	134
Patient 2 HDFs	D13S317	176
Patient 2 HDFs	D13S317	191
Patient 2 HDFs	D7S820	228
Patient 2 HDFs	D7S820	232
Patient 2 HDFs	D16S539	291
Patient 2 HDFs	D16S539	295
Patient 2 HDFs	CSF1PO	333
Patient 2 HDFs	CSF1PO	346
Patient 2 HDFs	Penta D	413
Patient 2 HDFs	Penta D	418
Patient 2 HDFs	AMEL	104
Patient 2 HDFs	AMEL	110
Patient 2 HDFs	vWA	146
Patient 2 HDFs	vWA	150
Patient 2 HDFs	D8S1179	234
Patient 2 HDFs	D8S1179	242
Patient 2 HDFs	TPOX	269
Patient 2 HDFs	TPOX	280
Patient 2 HDFs	FGA	346
Patient 2 HDFs	FGA	350

Sample File Name	Marker	Size
Patient 2 hiPSCs	D3S1358	124
Patient 2 hiPSCs	D3S1358	132
Patient 2 hiPSCs	TH01	162
Patient 2 hiPSCs	TH01	174
Patient 2 hiPSCs	D21S11	219
Patient 2 hiPSCs	D21S11	227
Patient 2 hiPSCs	D18S51	307
Patient 2 hiPSCs	D18S51	326
Patient 2 hiPSCs	Penta E	393
Patient 2 hiPSCs	Penta E	413
Patient 2 hiPSCs	D5S818	134
Patient 2 hiPSCs	D13S317	176
Patient 2 hiPSCs	D13S317	191
Patient 2 hiPSCs	D7S820	228
Patient 2 hiPSCs	D7S820	232
Patient 2 hiPSCs	D16S539	291
Patient 2 hiPSCs	D16S539	295
Patient 2 hiPSCs	CSF1PO	333
Patient 2 hiPSCs	CSF1PO	346
Patient 2 hiPSCs	Penta D	413
Patient 2 hiPSCs	Penta D	418
Patient 2 hiPSCs	AMEL	104
Patient 2 hiPSCs	AMEL	110
Patient 2 hiPSCs	vWA	146
Patient 2 hiPSCs	vWA	150
Patient 2 hiPSCs	D8S1179	234
Patient 2 hiPSCs	D8S1179	242
Patient 2 hiPSCs	TPOX	269
Patient 2 hiPSCs	TPOX	280
Patient 2 hiPSCs	FGA	346
Patient 2 hiPSCs	FGA	350

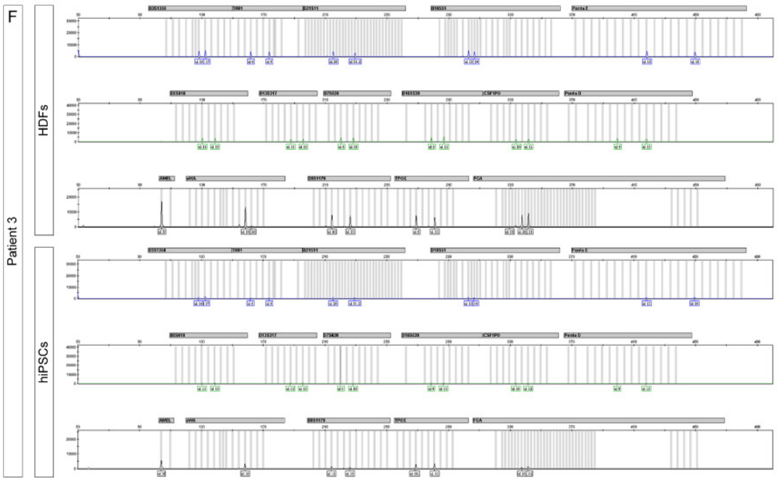

Sample File Name	Marker	Size
Patient 3 HDFs	D3S1358	128
Patient 3 HDFs	D3S1358	132
Patient 3 HDFs	TH01	162
Patient 3 HDFs	TH01	174
Patient 3 HDFs	D21S11	215
Patient 3 HDFs	D21S11	229
Patient 3 HDFs	D18S51	303
Patient 3 HDFs	D18S51	307
Patient 3 HDFs	Penta E	418
Patient 3 HDFs	Penta E	449
Patient 3 HDFs	D5S818	130
Patient 3 HDFs	D5S818	139
Patient 3 HDFs	D13S317	188
Patient 3 HDFs	D13S317	196
Patient 3 HDFs	D7S820	220
Patient 3 HDFs	D7S820	228
Patient 3 HDFs	D16S539	279
Patient 3 HDFs	D16S539	287
Patient 3 HDFs	CSF1PO	333
Patient 3 HDFs	CSF1PO	342
Patient 3 HDFs	Penta D	399
Patient 3 HDFs	Penta D	418
Patient 3 HDFs	AMEL	104
Patient 3 HDFs	vWA	158
Patient 3 HDFs	D8S1179	214
Patient 3 HDFs	D8S1179	226
Patient 3 HDFs	TPOX	269
Patient 3 HDFs	FGA	337
Patient 3 HDFs	FGA	342

Sample File Name	Marker	Size
Patient 3 hiPSCs	D3S1358	128
Patient 3 hiPSCs	D3S1358	132
Patient 3 hiPSCs	TH01	162
Patient 3 hiPSCs	TH01	174
Patient 3 hiPSCs	D21S11	215
Patient 3 hiPSCs	D21S11	229
Patient 3 hiPSCs	D18S51	303
Patient 3 hiPSCs	D18S51	307
Patient 3 hiPSCs	Penta E	418
Patient 3 hiPSCs	Penta E	449
Patient 3 hiPSCs	D5S818	130
Patient 3 hiPSCs	D5S818	139
Patient 3 hiPSCs	D13S317	188
Patient 3 hiPSCs	D13S317	196
Patient 3 hiPSCs	D7S820	220
Patient 3 hiPSCs	D7S820	228
Patient 3 hiPSCs	D16S539	279
Patient 3 hiPSCs	D16S539	287
Patient 3 hiPSCs	CSF1PO	333
Patient 3 hiPSCs	CSF1PO	342
Patient 3 hiPSCs	Penta D	399
Patient 3 hiPSCs	Penta D	418
Patient 3 hiPSCs	AMEL	104
Patient 3 hiPSCs	vWA	158
Patient 3 hiPSCs	D8S1179	214
Patient 3 hiPSCs	D8S1179	226
Patient 3 hiPSCs	TPOX	269
Patient 3 hiPSCs	FGA	337
Patient 3 hiPSCs	FGA	342

Figure S1. (**A**)-(**C**) Representative brightfield images of hiPSC clone morphology from all three HPDL patients. Scale Bars: 50 µm. (**D**)-(**F**) The images and tables, derived from STR based analysis, show the same identity between HPDL hiPSC lines and parental HDFs.

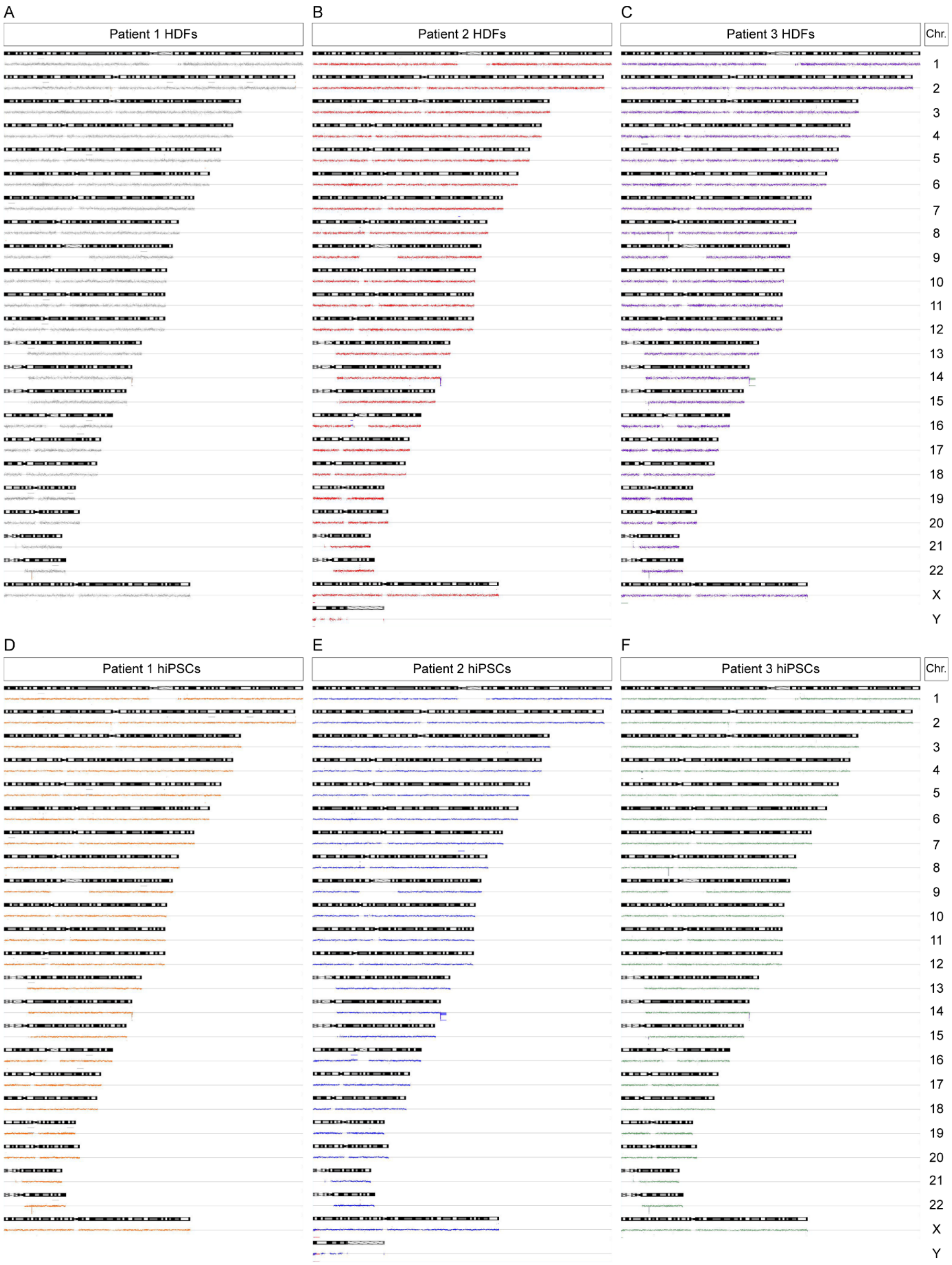

Figure S2. (**A**)-(**F**) The images, obtaining from aCGH analysis, indicate the integrity of our generated HPDL hiPSC lines, confirming even the identity with corresponding parental HDFs.

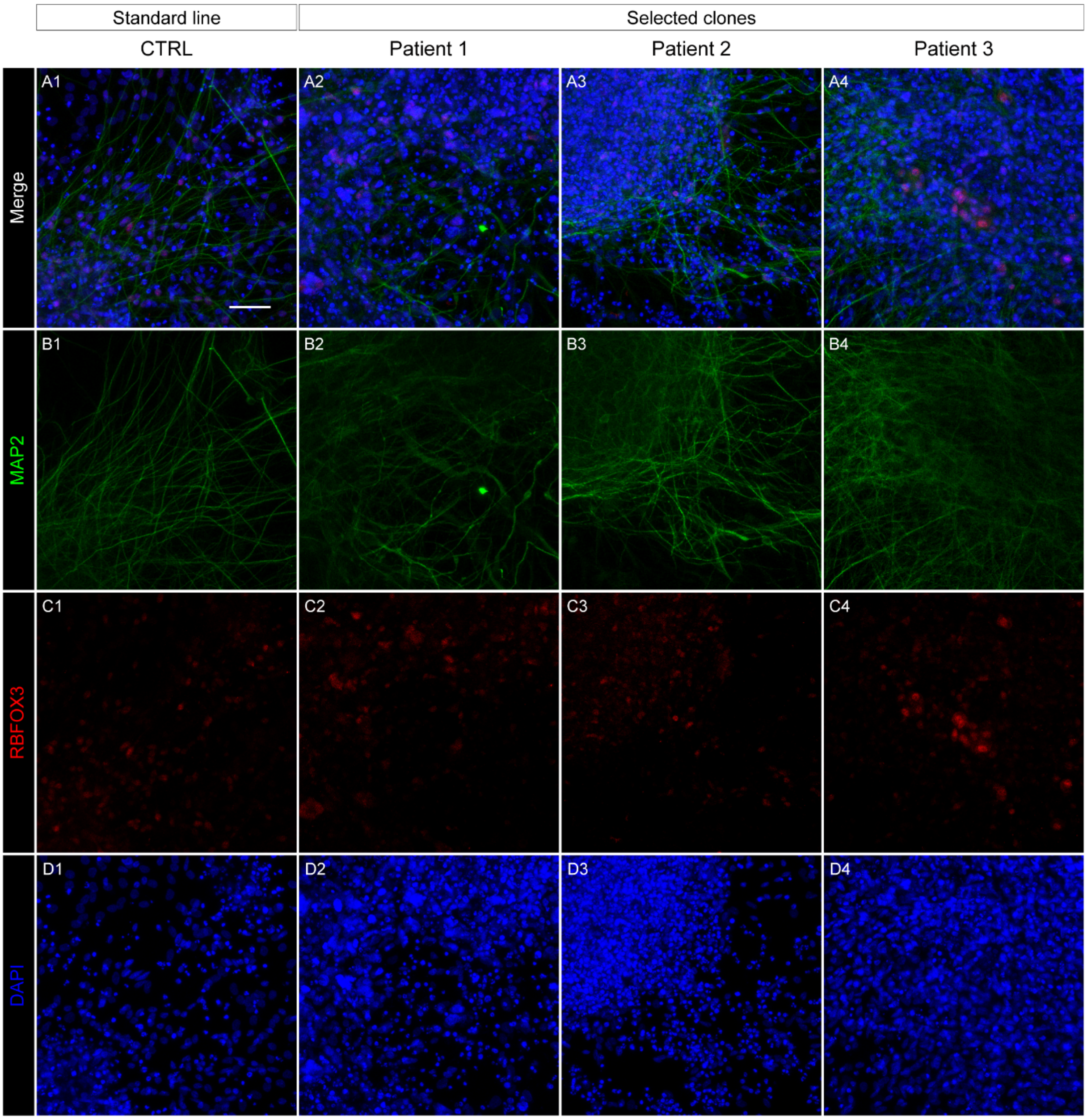

Figure S3. (**A**)-(**D**) Representative confocal images with neuronal markers MAP2 and RBFOX3 in all CTRL and HPDL neurons. All nuclei are marked with DAPI. Scale bar: 50 µm.
