# Supplementary material for: Generation and Characterization of hiPS Lines from Three Patients Affected by Different Forms of HPDL-Related Neurological Disorders
Source: Int J Mol Sci. 2024 Oct 2;25(19):10614. doi: 10.3390/ijms251910614 (PMC11477155; doi:10.3390/ijms251910614)
Supplement: Supplementary file 1 [file ijms-25-10614-s001.zip › Table S1.pdf]

Table S1. Summary of the clinical manifestations seen in each patient. UMN: upper motor neuron

Clinical features at skin biopsy age			
	**Patient 1**	**Patient 2**	**Patient 3**
Disease duration	12	15	3
Motor delay	Yes	Yes	No
Language delay	Yes	No	No
Intellectual disability	Yes (mild)	No	No
Spastic gait	Yes	Yes	Yes
Lower limb spasticity	Yes	Yes	Yes
Upper limb UMN involvement	Yes	Yes	No
Lower limb UMN involvement	Yes	Yes	Yes
Pseudobulbar signs	Yes	No	No
Bladder dysfunction	Yes	Yes	Yes
Ataxia	Yes	Yes	No
Extrapyramidal signs	Yes (hypokinetic syndrome)	No	No
Seizures	Yes (neonatal)	No	No
Oculomotor and visual abnormalities	Yes	Yes	No
Psychiatric manifestations	Yes (mood disorder and anxiety)	Yes (mood disorder and anxiety)	Yes (emotional immaturity and anxiety)
